# Supplementary material for: Recent increases in assemblage rarity are linked to increasing local immigration
Source: R Soc Open Sci. 2020 Jul 29;7(7):192045. doi: 10.1098/rsos.192045 (PMC7428220; doi:10.1098/rsos.192045)
Supplement: Supplimentary material Rarity Jones et al [file rsos192045supp1.docx]

Supplementary material

Contents

[Distribution map 1](#_Toc33093127)

[Number of Singletons 4](#_Toc33093128)

[Fisher’s Alpha 4](#_Toc33093129)

[Species Richness and Assemblage size 5](#_Toc33093130)

[Sensitivity testing 9](#_Toc33093131)

[Table S1. A list of all studies used in the analysis. 10](#_Toc33093132)

# Distribution map


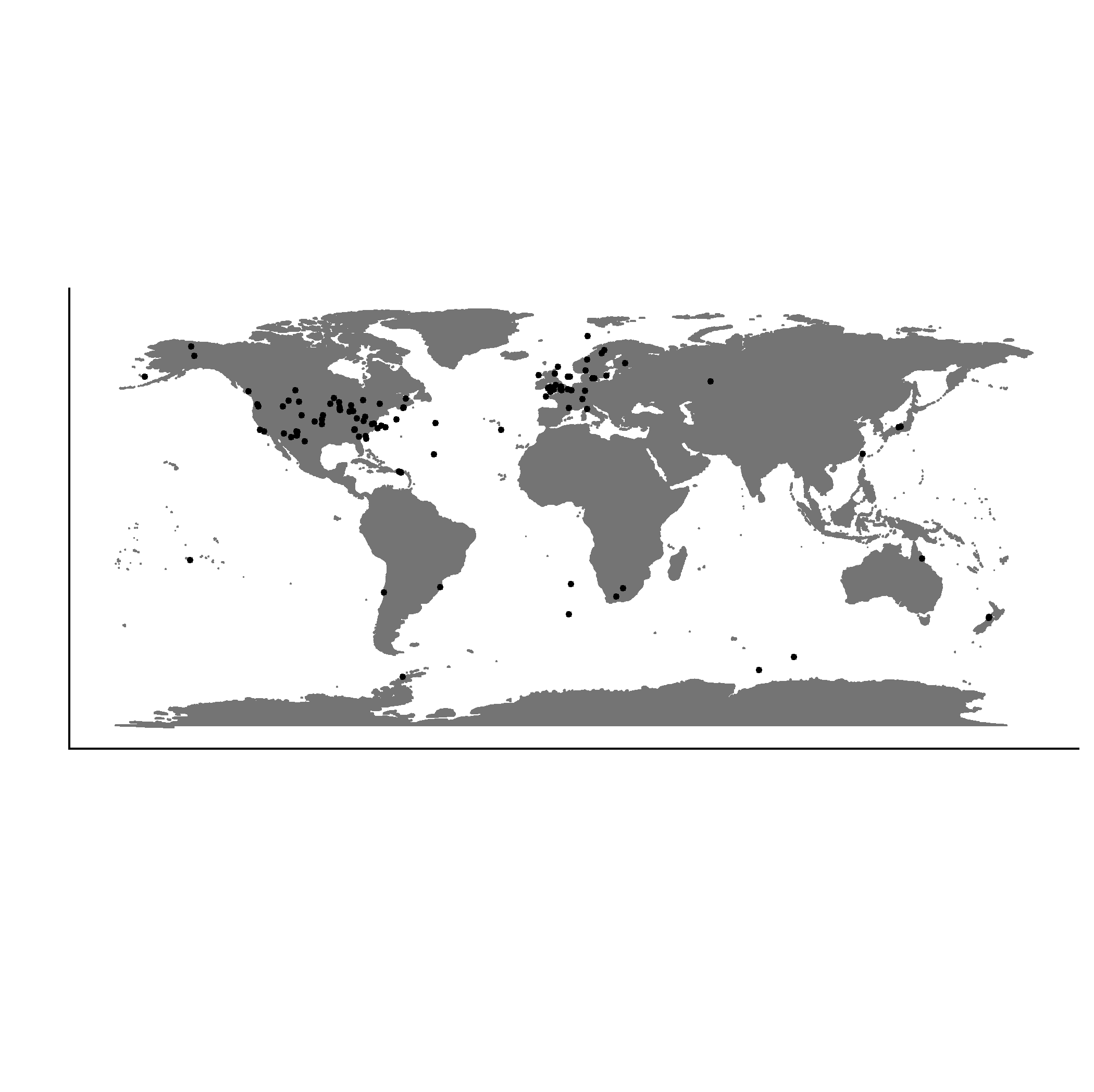


Figure S1. The central points of the 101 assemblages time series from the BioTIME database that are used in this study.


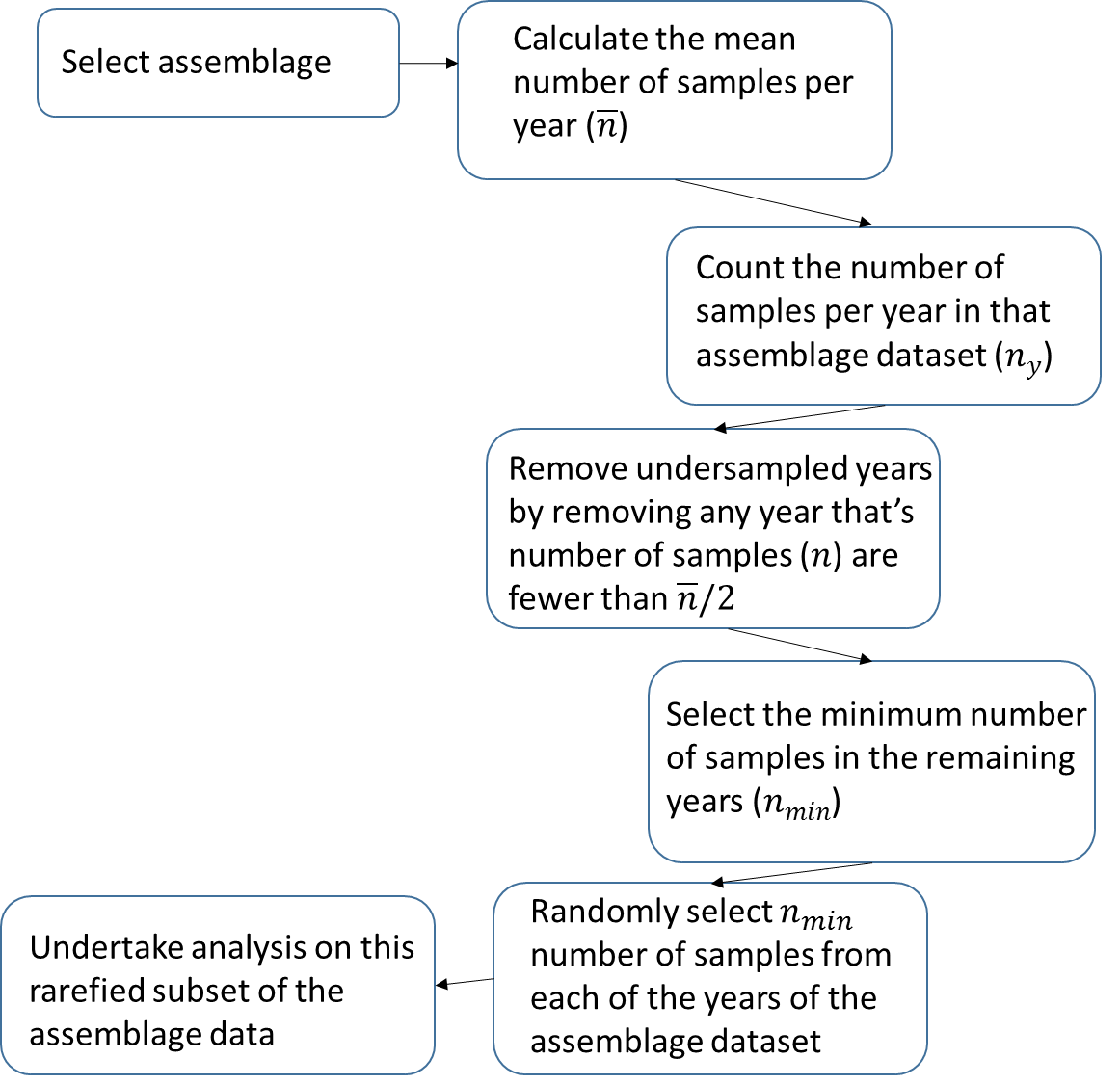


Figure S2. Workflow showing the process of rarefying a single assemblage in the BioTIME database.

| 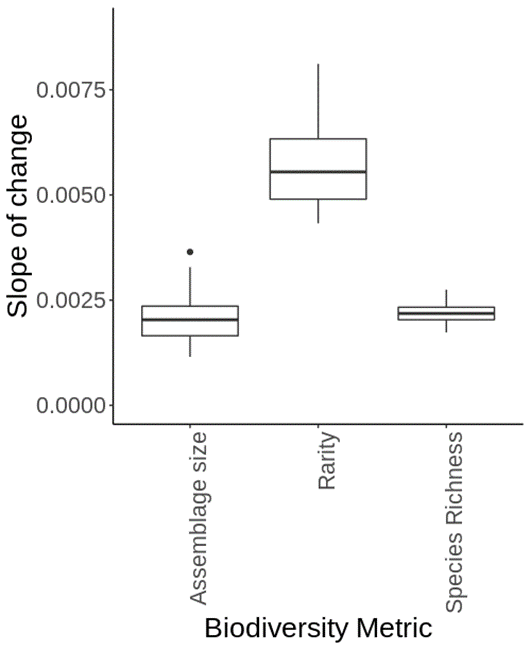 |
| --- |

Figure S3. The distribution of estimated slopes of change in numbers of rare species in terms of singletons and doubletons, species richness and assemblage size over time, as estimated 20 times after rarefaction. The positive results of the models were robust the variation introduced by rarefaction.

| 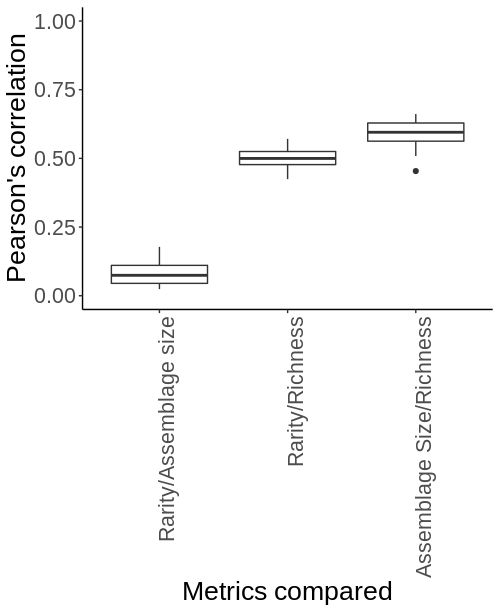 |
| --- |

Figure S4. The distribution of correlation values between slopes of change of rarity (singletons and doubletons), species richness and assemblage size across the results from the 20 different iterations of the rarefaction process. A positive relationship between rarity and species richness is evident in each iteration, so the result is robust to the variation introduced by the rarefaction process.

| 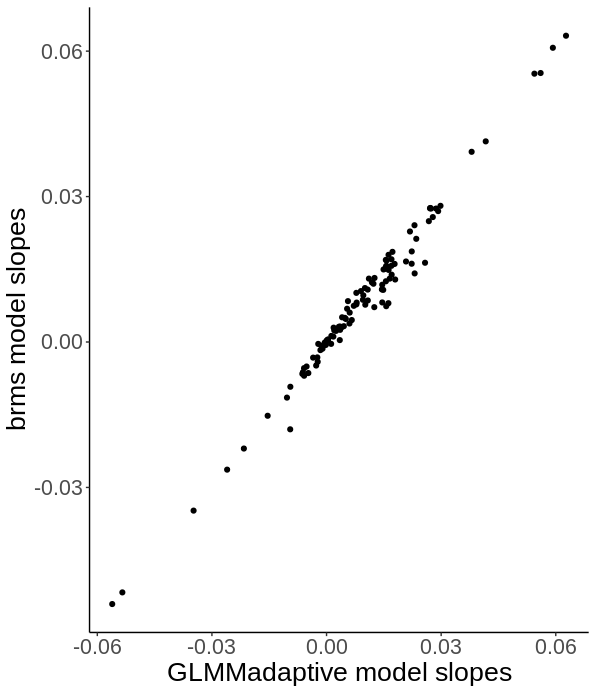 |
| --- |

Figure S5. The relationship between the estimated slopes of change in rarity for each assemblage as estimated by the brms and GLMMadaptive models.

| 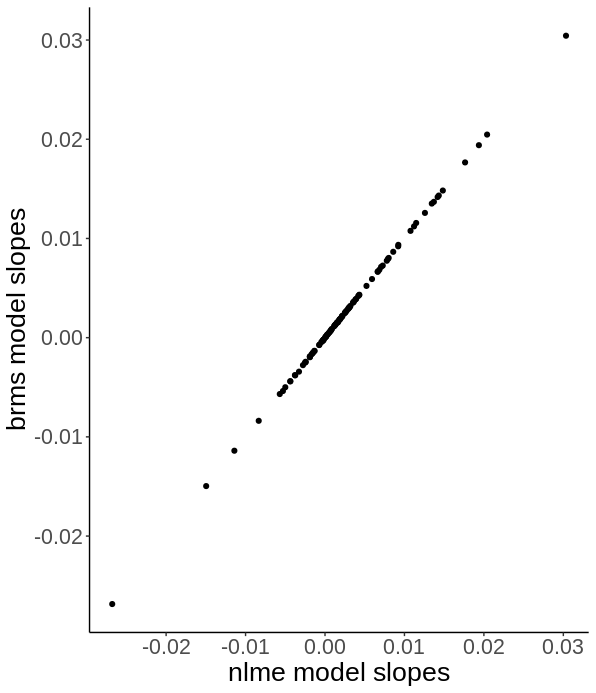 |
| --- |

Figure S6. The relationship between the estimated slopes of change in species richness for each assemblage as estimated by the brms and nlme models..

| 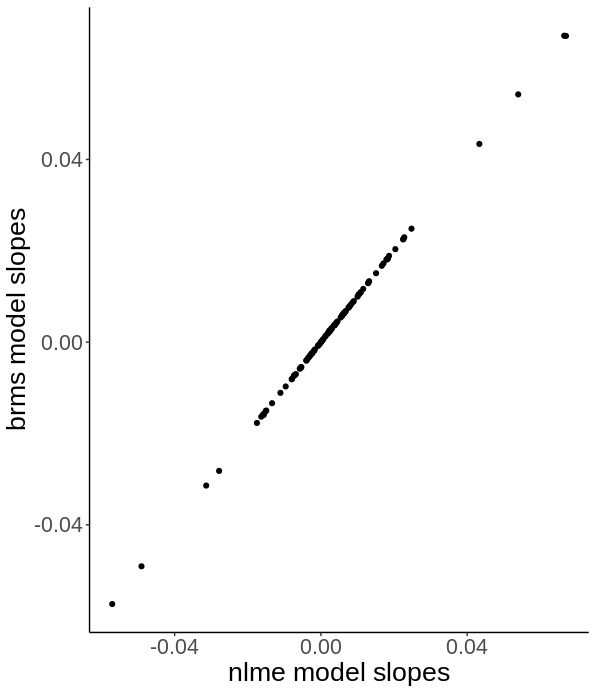 |
| --- |

Figure S7. The relationship between the estimated slopes of change in assemblage size for each assemblage as estimated by the brms and nlme models.

# Number of Singletons


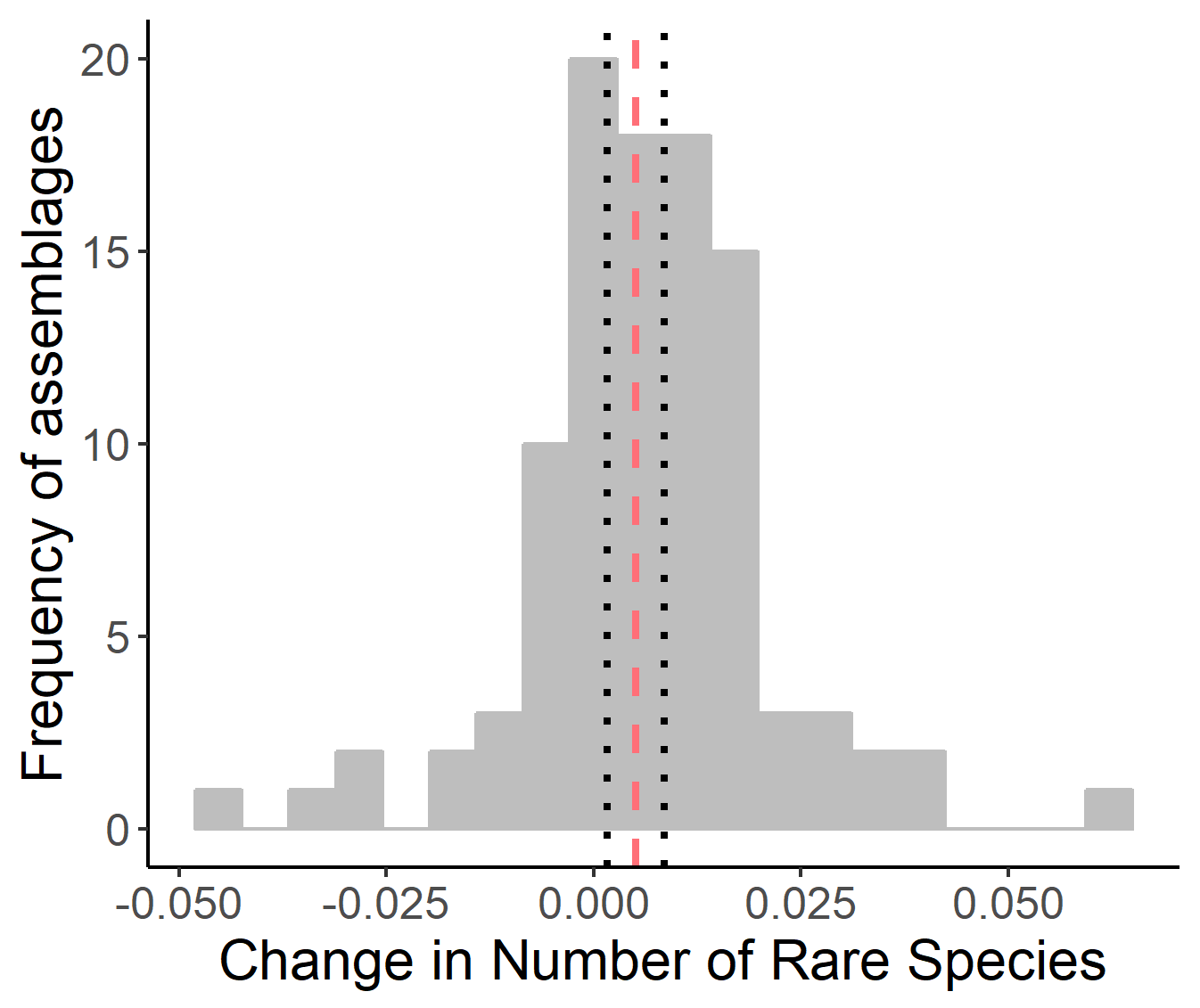


Figure S8. The distribution of slopes of change of the number of singletons within assemblages. The red vertical dashed line represents the overall global trend in the number of singletons, as calculated from the mixed model. The dotted black lines represent the standard error around this estimate.

# Fisher’s Alpha


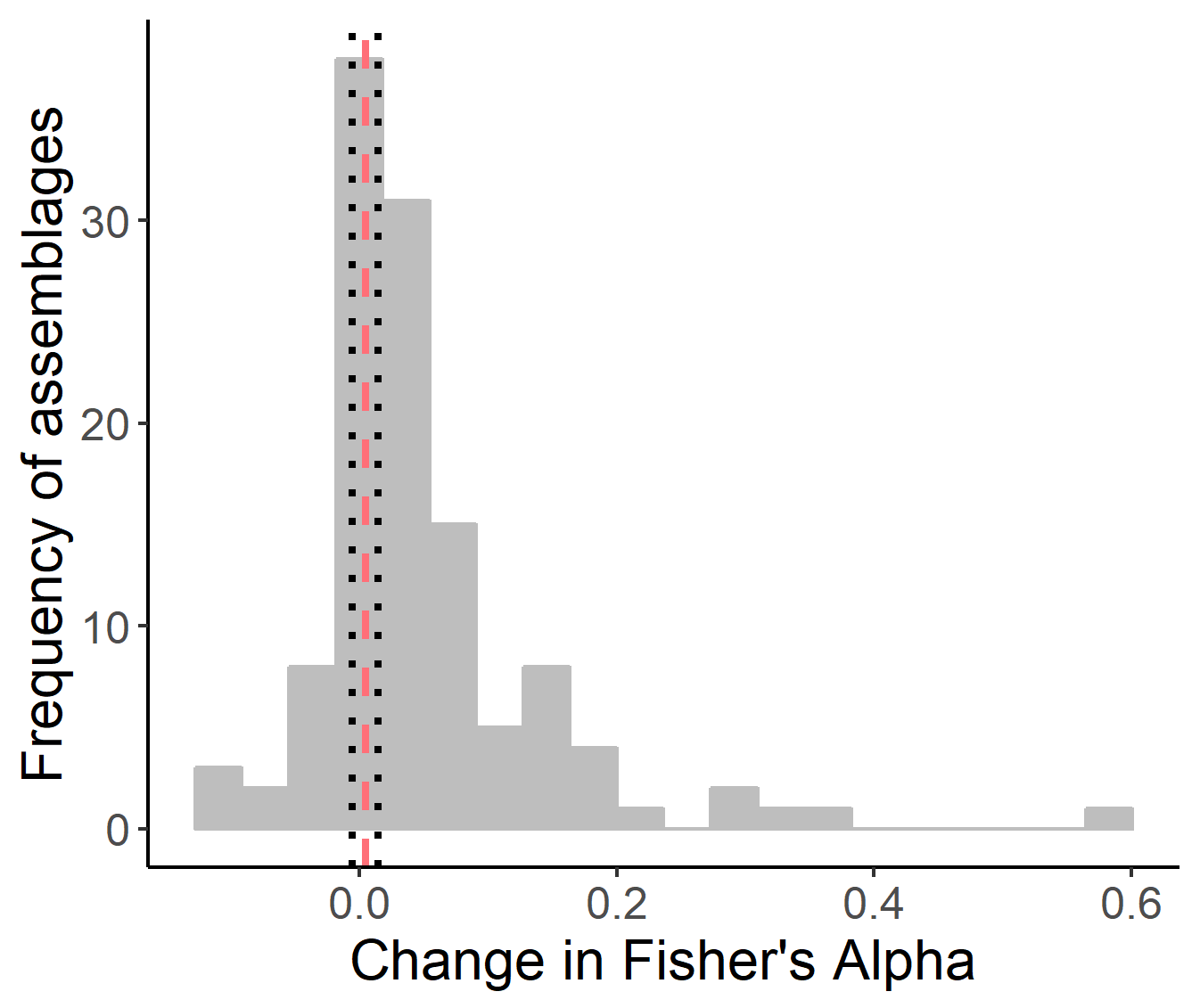


Figure S9. The distribution of slopes of change in Fisher’s Alpha. The red vertical dashed line represents the overall global trend in the number of singletons, as calculated from the mixed model. The dotted black lines represent the standard error around this estimate.

# Species Richness and Assemblage size


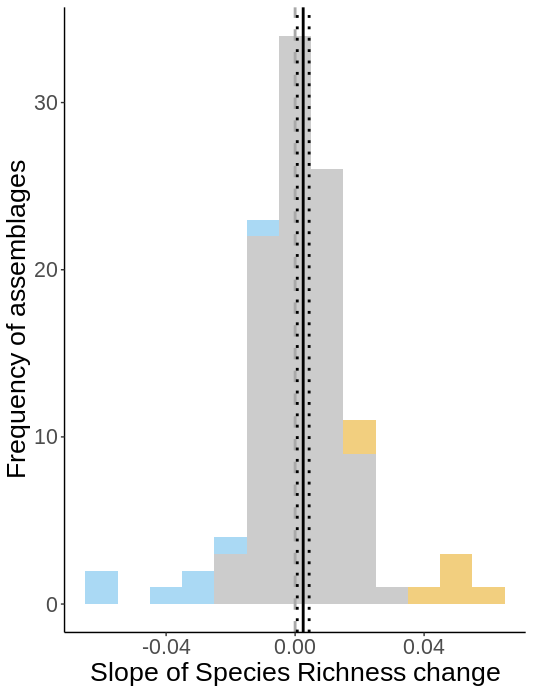


Figure S10. The distribution of slopes of change in assemblage species richness. Grey bars are where the 95% HPDI includes both positive and negative values, so the slope is not likely to be either positive or negative. The yellow bars are when the 95% HPDI of the slopes of change fall above 0, and the blue bars where the 95% HPDI of slopes of change fall below 0, so they represent assemblage slopes that are likely to be different from 0. The solid black line represents the mean overall global trend in changing numbers of rare species, and the dotted black lines represent the upper and lower 95% HPDI. The dashed grey line displays the 0 (no systematic trend) mark so that it is clear that the lower 95% HPDI of the main slope falls above 0 and so the model suggests a general increase in rarity.

| 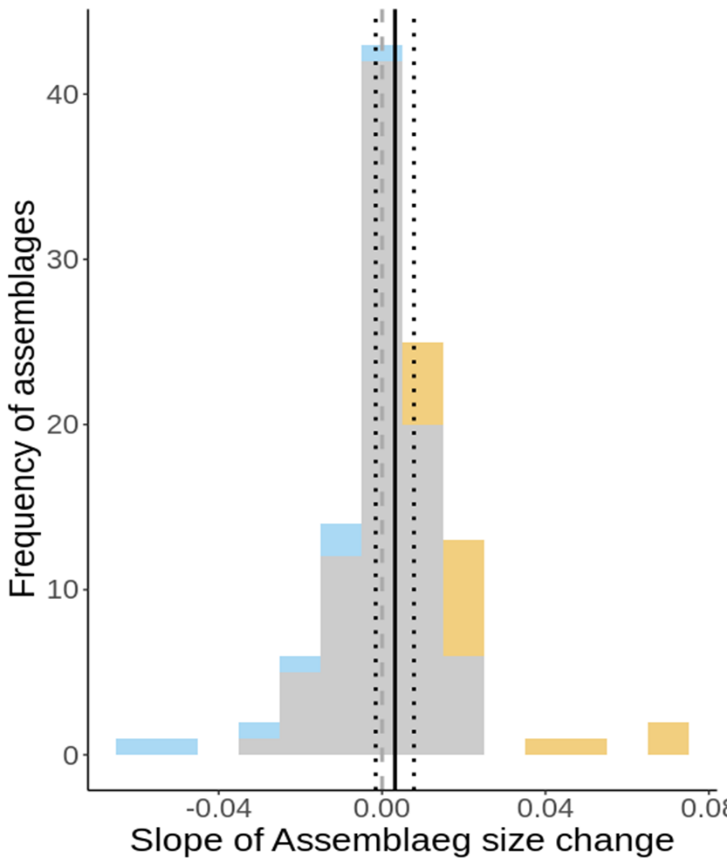 |
| --- |

Figure S11. The distribution of mean slopes of change in assemblage size. Grey bars are where the 95% HPDI includes both positive and negative values, so the slope is not likely to be either positive or negative. The yellow bars are when the 95% HPDI of the slopes of change fall above 0, and the blue bars where the 95% HPDI of slopes of change fall below 0, so they represent assemblage slopes that are likely to be different from 0. The solid black line represents the mean overall global trend in changing numbers of rare species, and the dotted black lines represent the upper and lower 95% HPDI. The dashed grey line displays the 0 (no systematic trend) mark, and shows that the lower 95% HPDI of the main slope falls slightly below 0 and so the model suggests a potential but not strong general increase in assemblage size.


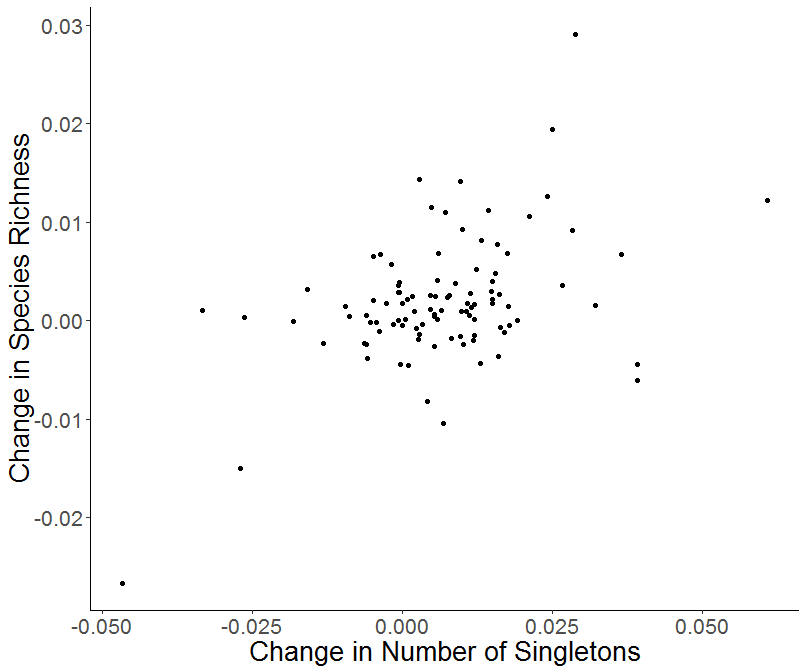


Figure S12. The relationship between rates of change the number of singletons and rates of change of species richness within assemblages. A positive relationship between these two community metrics is evident.


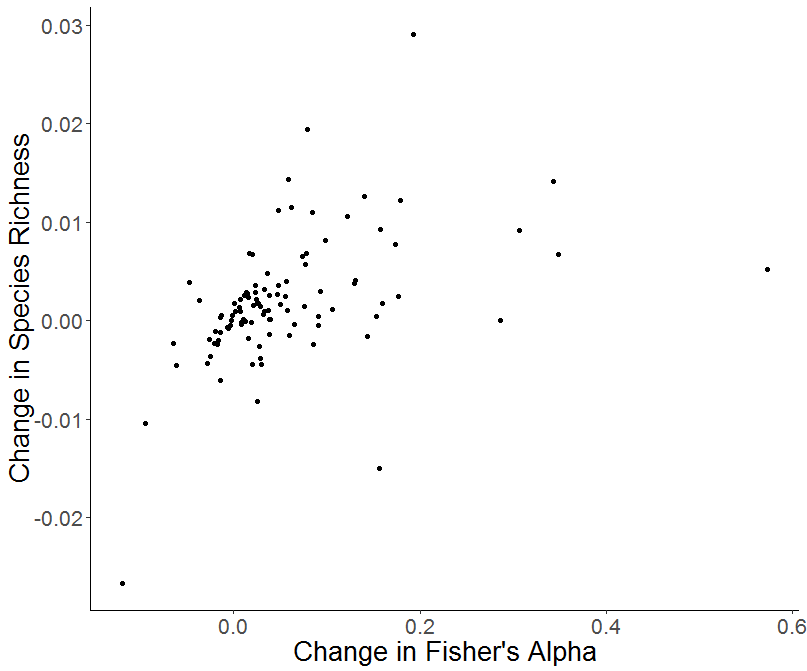


Figure S13. The relationship between rates of change of Fishers Alpha and rates of change of species richness within assemblages. The positive relationship between these two community metrics suggests that as Fisher’s Alpha increases, and so the proportion of rare species within assemblages increases, then species richness also increases.

# Sensitivity testing


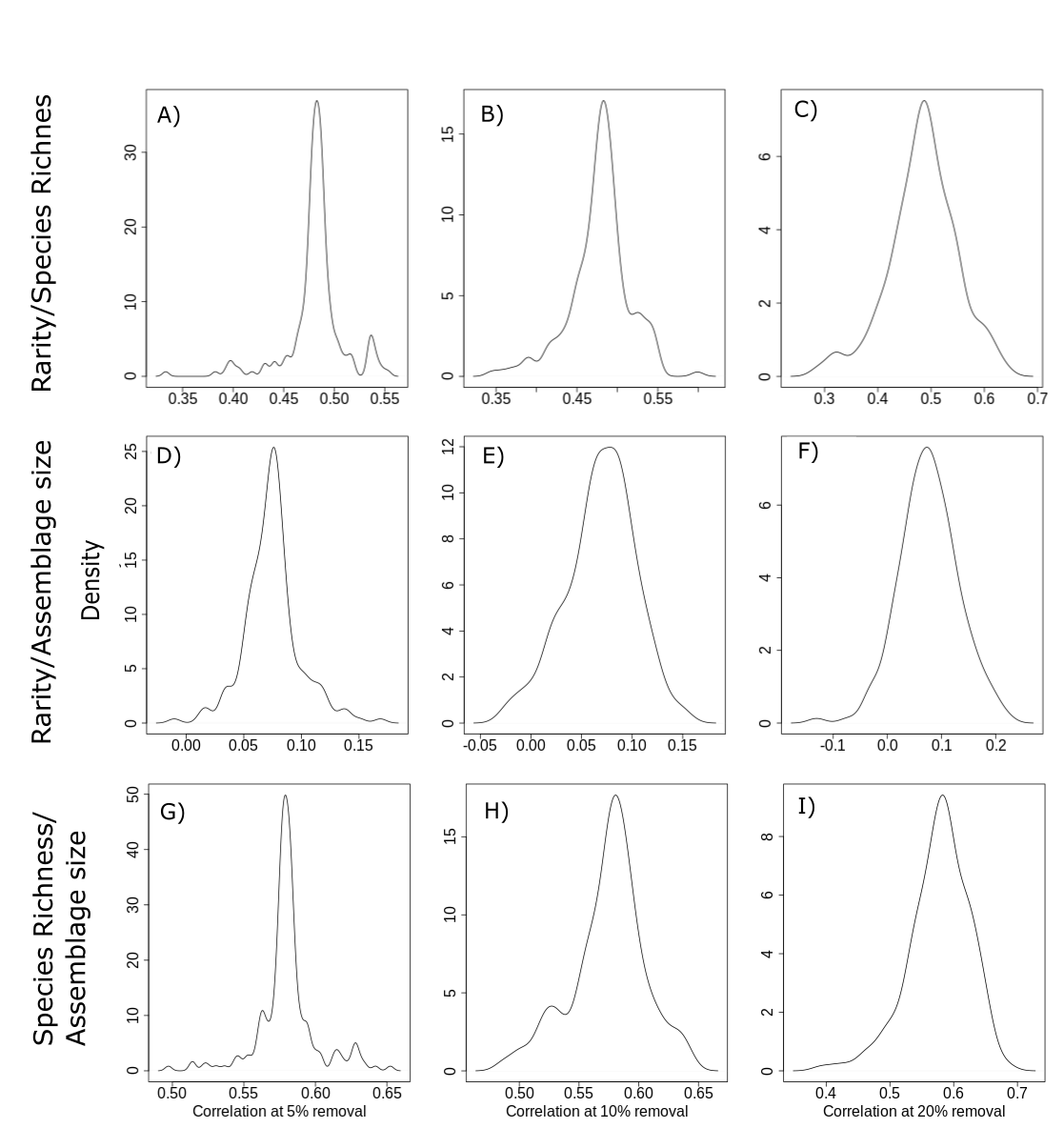


Figure S15. Testing the sensitivity of the relationship between species richness, rarity change (singletons and doubletons) and assemblage size within assemblages. We randomly removed a subset of 5%, 10% and 25% assemblages and then ran a Pearson’s Correlation test on the subset data. The uncertainty around the relationship increases when more assemblages are randomly removed, but the distribution of correlations remains consistently positive and centred around 0.45-0.5 for rarity and species richness, 0.07 for rarity and assemblage size and 0.55-0.6 for species richness and assemblage size.

# Table S1. A list of all studies used in the analysis.

These studies were sourced from the BioTIME database. This database is published as a data paper (Dornelas *et al*., 2018) and can be accessed from <http://biotime.st-andrews.ac.uk/home.php>

| Study ID | Realm | Climate | Taxa | Title | Number of years | Start year | End year | Central latitude | Central longitude | Number of Species | Total records | References |
| --- | --- | --- | --- | --- | --- | --- | --- | --- | --- | --- | --- | --- |
| 18 | Terrestrial | Temperate | Terrestrial plants | Mapped quadrats in sagebrush steppe long-term data for analyzing demographic rates and plant-plant interactions | 29 | 1923 | 1973 | 44.33 | -112.33 | 98 | 8034 | Zachmann L, Moffet C, Adler P. Mapped quadrats in sagebrush steppe: long‐term data for analyzing demographic rates and plant–plant interactions. Ecology. 2010;91(11):3427-. |
| 41 | Terrestrial | Temperate | Birds | Time and space and the variation of species | 10 | 1923 | 1940 | 39.5 | -82.48 | 56 | 418 | Preston FW. Time and space and the variation of species. Ecology. 1960;41(4):611-27. |
| 42 | Terrestrial | Temperate | Birds | Eastern Wood | 30 | 1949 | 1979 | 51.2965 | -0.38352 | 45 | 954 | Gaston KJ, Blackburn TM. Pattern and process in macroecology. Oxford, England: Wiley-Blackwell; 2000.  Beven G. Changes in breeding bird populations of an oak-wood on Bookham Common, Surrey, over twenty-seven years. London Naturalist. 1976;55:23-42.  Gibbons DW, Reid JB, Chapman RA. The new atlas of breeding birds in Britain and Ireland: 1988-1991: T & AD Poyser London; 1993.  Stone B, Sears J, Cranswick P, Gregory R, Gibbons D, Rehfisch M, *et al*. Population estimates of birds in Britain and in the United Kingdom. British Birds. 1997;90(1):1-22.  Lack P. The atlas of wintering birds in Britain and Ireland: A&C Black; 2010.  Standley P, Bucknell N, Swash A, Collins I. The Birds of Berkshire. Berkshire Atlas Group. Reading, UK. 1996.  Williamson M, editor Are communities ever stable? Symposium of the British Ecological Society; 1987. |
| 44 | Terrestrial | Temperate | Terrestrial plants | Plant succession and biomass dynamics following logging and burning in the Andrews Experimental Forest Watersheds 1 and 3. 1962-Present | 16 | 1962 | 1977 | 44.33 | -122.33 | 158 | 25571 | Halpern CB, Dyrness C. "Plant succession and biomass dynamics following logging and burning in the Andrews Experimental Forest Watersheds 1 and 3, 1962-Present". Long-Term Ecological Research. Forest Science Data Bank, Corvallis. 2010;  Halpern CB, Lutz JA. Canopy closure exerts weak controls on understory dynamics: a 30‐year study of overstory–understory interactions. Ecological Monographs. 2013;83(2):221-37.  Halpern CB, Lutz JA. "Canopy closure exerts weak controls on understory dynamics: a 30‐year study of overstory–understory interactions". 2013;Available at: Dryad DigitalRepository, doi:10.5061/dryad.1q88j:accessed 2013. |
| 46 | Terrestrial | Temperate | Birds | Skokholm Bird Observatory | 47 | 1928 | 1979 | 51.698 | -5.277 | 29 | 528 | Williamson M. The land-bird community of Skokholm: ordination and turnover. Oikos. 1983:378-84. |
| 47 | Terrestrial | Temperate | Birds | Detection of Density-Dependent Effects in Annual Duck Censuses | 26 | 1952 | 1977 | 50.84545 | -107.446 | 13 | 392 | Vickery WL, Nudds TD. Detection of Density‐Dependent Effects in Annual Duck Censuses. Ecology. 1984;65(1):96-104. |
| 54 | Terrestrial | Tropical | Terrestrial invertebrates | El Verde Grid invertebrate data (Big Grid Snail Captures 1991-2007) | 24 | 1991 | 2014 | 18.1667 | -65.5 | 19 | 21702 | Willig MRB, C. P. . "El Verde Grid long-term invertebrate data: Luquillo Long Term Ecological Research Site Database: Data Set 107". 2016;Available at:http://luq.lternet.edu/data/luqmetadata107/7427 accessed 2016. |
| 56 | Terrestrial | Temperate | Mammals | Small Mammal Mark-Recapture Population Dynamics at Core Research Sites | 20 | 1989 | 2008 | 34.2 | -106.43 | 28 | 16657 | Friggens M. "Sevilleta LTER Small Mammal Population Data", Albuquerque, NM: Sevilleta Long Term Ecological Research Site Database: SEV008. 2008;Available at: http://sev.lternet.edu/data/sev-8:accessed 2012. |
| 57 | Freshwater | Temperate | Fish | North Temperate Lakes LTER Fish Abundance | 32 | 1981 | 2012 | 43.9928 | -89.4946 | 76 | 10892 | LTER N. "NTLFI02 North Temperate Lakes LTER: Fish Abundance 1981 - current". North Temperate Lakes Long Term Ecological Research program, NSF. Center for Limnology, University of Wisconsin-Madison.Available at: https://lter.limnology.wisc.edu/dataset/north-temperate-lakes-lter-fish-abundance-1981-current:accessed 2012. |
| 58 | Terrestrial | Tropical | Birds | Avian populations long-term monitoring dataset. San Juan. Puerto Rico Luquillo Long Term Ecological Research Site Database Grid points bird counts DBAS 23 | 18 | 1991 | 2008 | 18.19 | -65.43 | 31 | 1171 | Waide RB. Bird abundance - point counts. Long Term Ecological Research Network. 2010;Available at: http://dx.doi.org/10.6073/pasta/0d96957379936a038ebbbcc6135b2fab:accessed 2012.  Waide RB. "Bird abundance - point counts. El Verde Field Station, Puerto Rico: Luquillo Long Term Ecological Research Site Database: Data Set 23". 2010;Available at: http:/luq.lter net.edu/data/luqmetadata23:accessed 2012. |
| 59 | Terrestrial | Temperate | Mammals | Long-term monitoring and experimental manipulation of a Chihuahuan Desert ecosystem near Portal. Arizona. USA | 26 | 1977 | 2002 | 30.3226 | -103.501 | 29 | 427 | Ernest S, Valone TJ, Brown JH. Long‐term monitoring and experimental manipulation of a Chihuahuan Desert ecosystem near Portal, Arizona, USA. Ecology. 2009;90(6):1708-. |
| 67 | Terrestrial | Temperate | Birds | Animal Demography Unit - Coordinated Waterbird Counts (CWAC) (AfrOBIS) | 24 | 1983 | 2006 | -28.9545 | 24.95096 | 68 | 15448 | CWAC. "Coordinated Waterbird Counts (CWAC) - AfrOBIS". .Available at: http://www.iobis.org/mapper/?dataset=603:accessed 2012. |
| 81 | Marine | Temperate | Mammals | CRRU Cetacean sighting in Scotland waters 1997-2010 | 14 | 1997 | 2010 | 57.76701 | -2.643 | 9 | 1613 | Robinson KP. "CRRU (Cetacean Research and Rescue Unit) Cetacean sightings in Scotland waters". 2010;Available at: http://www.emodnet-biology.eu/component/imis/?module=dataset&dasid=2819:accessed 2012.  Robinson KP, Baumgartner N, Eisfeld SM, Clark NM, Culloch RM, Haskins GN, *et al*. The summer distribution and occurrence of cetaceans in the coastal waters of the outer southern Moray Firth in northeast Scotland (UK). Lutra. 2007;50(1):19. |
| 97 | Marine | Polar/Temperate | Marine invertebrates | Archives of the Arctic Seas Zooplankton (ARC) | 22 | 1921 | 1973 | 72.73944 | 10.69445 | 402 | 15016 | Markhaseva EL, Golikov AA, Agapova TA, Beig AA. Archives of the Arctic Seas Zooplankton. 1985;Available at: http://www.iobis.org/mapper/?dataset=4470:accessed 2012. |
| 100 | Marine | Temperate | Fish | Community level response to climate change The long-term study of the fish andcrustacean community of the Bristol Channel | 31 | 1981 | 2011 | 51.14 | -3.08 | 83 | 5199 | Henderson PA. The long-term study of the fish and crustacean community of the Bristol Channel.Available at http://www.pisces-conservation.com/:accessed 2013.  Henderson PA, Magurran AE. Direct evidence that density-dependent regulation underpins the temporal stability of abundant species in a diverse animal community. Proceedings of the Royal Society B: Biological Sciences. 2014;281(1791).  Henderson PA, Magurran AE. Data from: Direct evidence that density-dependent regulation underpins the temporal stability of abundant species in a diverse animal community. Dryad Data Repository; 2014. |
| 108 | Marine | Global | Birds | Seabirds of the Southern and South Indian Ocean (Australian Antarctic Data Centre) | 29 | 1977 | 2006 | -27.1736 | 3.945813 | 123 | 116226 | Woehler E. "Seabirds of the Southern and South Indian Ocean - Australian Antarctic Data Centre". Available at: http://www.iobis.org:accessed 2012. |
| 112 | Marine | Temperate/Tropical | Fish | NOAA Southeast Fishery Science Center (SEFSC) Commercial Pelagic Observer Program (POP) Data (SEFSC_POP) | 22 | 1973 | 2005 | 24.98149 | -51.3741 | 540 | 466437 | NIWA. "South Western Pacific Regional OBIS Data Asteroid Subset", NIWA (National Institute of Water and Atmospheric Research - New Zealand) MBIS (Marine Biodata Information System) accessed through South Western Pacific OBIS.;Available at: http://www.iobis.org/mapper/?dataset=219:accessed 2012. |
| 117 | Marine | Temperate | Marine invertebrates | South Western Pacific Regional OBIS Data Asteroid Subset (South Western Pacific OBIS) | 44 | 1956 | 2003 | -39.3717 | 3.081897 | 156 | 2253 | NODC. "South TX Outer Continental Shelf and MI, AL, and FL Outer Continental Shelf benthic organism sampling 1974-1978". US National Oceanographic Data Center, Silver Spring, Maryland, USA. 2011;Available at http://www.usgs.gov/obis-usa/data_search_and_access/participants.html:accessed 2012. |
| 119 | Marine | Temperate | Fish | DFO Maritimes Research Vessel Trawl Surveys Fish Observations (OBIS Canada) | 41 | 1970 | 2010 | 43.98743 | -63.6697 | 231 | 121804 | Clark D, Branton B. DFO Maritimes Research Vessel Trawl Surveys, OBIS Canada Digital Collections. Bedford Institute of Oceanography, Dartmouth, Nova Scotia, Canada, OBIS Canada. 2007. |
| 125 | Marine | Temperate | Fish | MARMAP Chevron Trap Survey 1990-2009 (OBIS-USA) | 12 | 1988 | 2000 | 31.44165 | -78.8489 | 101 | 15092 | Reichert M. "MARMAP Chevron Trap Survey 1990-2009". SCDNR/NOAA MARMAP Program, SCDNR MARMAP Aggregate Data Surveys, The Marine Resources Monitoring, Assessment, and Prediction (MARMAP) Program, Marine Resources Research Institute, South Carolina Department of Natural Resources U.S.A. 2009;Available at: http://www.usgs.gov/obis-usa/data_search_and_access/participants.html:accessed 2012. |
| 163 | Marine | Temperate | Benthos | North Pacific Groundfish Observer (North Pacific Research Board) | 12 | 1993 | 2004 | 56.5 | -168.15 | 355 | 419940 | NPRB. "The Observer Program database", accessed through the OBIS-USA North Pacific Groundfish Observer (North Pacific Research Board). Available at: http://www.iobis.org:accessed 2012. . |
| 166 | Marine | Global | All | PIROP Northwest Atlantic 1965-1992 (SEAMAP) | 25 | 1965 | 1992 | 36.07524 | -70.9918 | 213 | 155600 | Diamond A, Gaston A, Brown R. Converting PIROP Counts of Seabirds at Sea to Absolute Densities. Progress Notes No 164. Canadian Wildlife Service, Ottawa. 1986.  Huettmann F. An ecological GIS research application for the northern Atlantic-The PIROP database software, environmental data sets and the role of the internet. Riekert/Tochtermann. 1998.  PIROP. "PIROP Northwest Atlantic 1965-1992 - OBIS SEAMAP".Available at: http://www.iobis.org/mapper/?dataset=2245:accessed 2012.  Brown RG. Atlas of eastern Canadian seabirds. 1975.  Halpin PN, Read AJ, Fujioka E, Best BD, Donnelly B, Hazen LJ, *et al*. OBIS-SEAMAP: The world data center for marine mammal, sea bird, and sea turtle distributions. Oceanography. 2009;22(2):104-15.  Read A, Halpin P, Crowder L, Best B, Fujioka E. OBIS-SEAMAP: mapping marine mammals, birds and turtles. World Wide Web electronic publication http://seamap env duke edu Accessed. 2009;15. |
| 169 | Marine | Temperate | All | CalCOFI and NMFS Seabird and Marine Mammal Observation Data. 1987-2006 (SEAMAP) | 20 | 1987 | 2006 | 34.85846 | -121.615 | 185 | 61730 | Jahncke JR, C. "CalCOFI and NMFS Seabird and Marine Mammal Observation Data, 1987-2006". California Cooperative Oceanic Fisheries Investigations (CalCOFI) and National Marine Fisheries Service (NMFS) cruises, 1987-2006 - OBIS SEAMAP. 2006;Available at: http://www.iobis.org:accessed 2012.  Rintoul C, Langabeer-Schlagenhauf B, Hyrenbach K, Morgan K, Sydeman W. Atlas of California Current marine birds and mammals: Version 1. Unpublished Report, PRBO Conservation Science, Petaluma, CA. 2006.  Yen P, Sydeman W, Bograd S, Hyrenbach K. Spring-time distributions of migratory marine birds in the southern California Current: Oceanic eddy associations and coastal habitat hotspots over 17 years. Deep Sea Research Part II: Topical Studies in Oceanography. 2006;53(3):399-418.  Yen PP, Sydeman WJ, Hyrenbach KD. Marine bird and cetacean associations with bathymetric habitats and shallow-water topographies: implications for trophic transfer and conservation. Journal of Marine Systems. 2004;50(1):79-99. |
| 172 | Marine | Temperate | All | POPA cetacean. seabird. and sea turtle sightings in the Azores area 1998-2009 (OBIS SEAMAP) | 12 | 1998 | 2009 | 35.00974 | -24.2247 | 47 | 52291 | POPA. "cetacean, seabird, and sea turtle sightings in the Azores area 1998-2009 - OBIS SEAMAP". .Available at: http://www.iobis.org/mapper/?dataset=4257:accessed 2012.  Amorim P, Figueiredo M, Machete M, Morato T, Martins A, Serrão Santos R. Spatial variability of seabird distribution associated with environmental factors: a case study of marine Important Bird Areas in the Azores. ICES Journal of Marine Science. 2008;66(1):29-40.  Machete M, Santos R, editors. Azores Fisheries Observer Program (POPA): a case study of the multidisciplinary use of observer data. Proceedings of the 5th International Fisheries Observer Conference; 2007.  Morato T, Varkey DA, Damaso C, Machete M, Santos M, Prieto R, *et al*. Evidence of a seamount effect on aggregating visitors. Marine Ecology Progress Series. 2008;357:23-32. |
| 180 | Marine | Polar/Temperate | Fish | ECNASAP - East Coast North America Strategic Assessment (OBIS Canada) | 26 | 1970 | 1995 | 37.77056 | -50.7927 | 273 | 410802 | ECNA. "East Coast North America Strategic Assessment Project, Groundfish Atlas for the East Coast of North America".Available at: http://www.iobis.org:accessed 2012. |
| 182 | Marine | Temperate | All | Snow crab research trawl survey database (Southern Gulf of St. Lawrence. Gulf region. Canada) from 1988 to 2010 (OBIS Canada) | 22 | 1988 | 2009 | 47.48092 | -62.7617 | 33 | 35005 | Wade E. Snow crab research trawl survey database (Southern Gulf of St. Lawrence, Gulf region, Canada) from 1988 to 2010. OBIS Canada, Bedford Institute of Oceanography, Dartmouth, Nova Scotia, Canada. 2011. |
| 183 | Marine | Temperate | Marine invertebrates | DFO Maritimes Research Vessel Trawl Surveys Invertebrate Observations (OBIS Canada) | 13 | 1999 | 2011 | 43.77665 | -63.7514 | 16 | 14906 | Tremblay JM, Branton B. DFO Maritimes Research Vessel Trawl Surveys, OBIS Canada Digital Collections. Bedford Institute of Oceanography, Dartmouth, Nova Scotia, Canada, OBIS Canada. 2007. |
| 190 | Marine | Tropical | Fish | St. Croix. USVI Fish Assessment and Monitoring Data (2002 - Present) (NOAA-CCMA) | 10 | 2001 | 2010 | 17.75697 | -64.6043 | 247 | 28017 | USVI. "St. Croix, USVI Fish Assessment and Monitoring Data (2002 - Present)", Silver Spring, MD Publisher: NOAAs Ocean Service, National Centers for Coastal Ocean Science (NCCOS). National Oceanic and Atmospheric Association (NOAA)-National Ocean Service (NOS)-National Centers for Coastal Ocean Science (NCCOS)-Center for Coastal Monitoring and Assessment (CCMA)-Biogeography Team. 2007;Available at: http://www.iobis.org/mapper/?dataset=1673:accessed 2012. |
| 191 | Marine | Temperate | Marine invertebrates | NEFSC Benthic Database (OBIS-USA) | 28 | 1900 | 1975 | 39.12306 | -66.6414 | 1614 | 51456 | NEFSC. "Benthic Database (OBIS-USA)", Northeast Fisheries Science Center, National Marine Fisheries Service, NOAA, U.S. Department of Commerce. 2010;Available at: http://www.iobis.org/mapper/?dataset=1694:accessed 2012. |
| 195 | Terrestrial | Temperate | Birds | Breeding birds survey North America | 30 | 1978 | 2007 | 40.80924 | -96.1873 | 385 | 699449 | USGS. Patuxent Wildlife Research Center “North American Breeding Bird Survey” ftp data set, version 2014.0.Available at: ftp://ftpext.usgs.gov/pub/er/md/laurel/BBS/DataFiles/:accessed 2013. |
| 196 | Marine | Temperate | Benthos | SOTEAG Rocky Shore Survey (Sullom Voe) | 35 | 1976 | 2012 | 60.46645 | -1.32288 | 252 | 91491 | Moore JJ, Howson CM. “Survey of the rocky shores in the region of Sullom Voe, Shetland, A report to SOTEAG from Aquatic Survey & Monitoring Ltd”, Cosheston, Pembrokeshire. 29 p.Available at: http://www.soteag.org.uk:accessed 2013. |
| 200 | Marine | Temperate | Marine invertebrates | NEFSC Benthic Database (OBIS-USA) | 30 | 1956 | 1989 | 35.69171 | -74.0908 | 2105 | 102143 | DATRAS. "Fish trawl survey: Northern Irish Ground Fish Trawl Survey. ICES Database of trawl surveys (DATRAS)." The International Council for the Exploration of the Sea, Copenhagen. 2010;Available at: http://www.emodnet-biology.eu/data-catalog?%3Fmodule=dataset&dasid=2764:accessed 2013. |
| 206 | Marine | Temperate | Fish | Northern Irish Ground Fish Trawl Survey | 16 | 1993 | 2008 | 52.17598 | -4.62847 | 108 | 13547 | DATRAS. "Fish trawl survey: Northern Irish Ground Fish Trawl Survey. ICES Database of trawl surveys (DATRAS)." The International Council for the Exploration of the Sea, Copenhagen. 2010;Available at: http://www.emodnet-biology.eu/data-catalog?%3Fmodule=dataset&dasid=2764:accessed 2013. |
| 208 | Marine | Temperate | Fish | ICES French Southern Atlantic Bottom Trawl Survey for commercial fish species. ICES Database of trawl surveys (DATRAS) | 11 | 1997 | 2007 | 48.52178 | -6.24898 | 189 | 24867 | DATRAS. "Fish trawl survey: ICES French Southern Atlantic Bottom Trawl Survey for commercial fish species. ICES Database of trawl surveys (DATRAS)." The International Council for the Exploration of the Sea, Copenhagen. 2010;Available at: http://www.emodnet-biology.eu/data-catalog?%3Fmodule=dataset&dasid=2759:accessed 2013. |
| 209 | Marine | Temperate | Fish | ICES Beam Trawl Survey for commercial fish species. ICES Database of trawl surveys (DATRAS) -UK | 18 | 1990 | 2007 | 51.09476 | 0.085737 | 115 | 19711 | DATRAS. "Fish trawl survey: ICES Beam Trawl Survey for commercial fish species. ICES Database of trawl surveys (DATRAS)." The International Council for the Exploration of the Sea, Copenhagen. 2010;Available at: http://www.emodnet-biology.eu/data-catalog?%3Fmodule=dataset&dasid=2761:accessed 2013. |
| 210 | Marine | Temperate | Fish | ICES North Sea International Bottom Trawl Survey for commercial fish species. ICES Database of trawl surveys (DATRAS) | 47 | 1965 | 2011 | 56.46378 | 3.500367 | 254 | 296524 | DATRAS. "Fish trawl survey: ICES North Sea International Bottom Trawl Survey for commercial fish species. ICES Database of trawl surveys (DATRAS)." The International Council for the Exploration of the Sea, Copenhagen. 2010;Available at: http://www.emodnet-biology.eu/data-catalog?%3Fmodule=dataset&dasid=2763:accessed 2013. |
| 213 | Marine | Temperate | Benthos | Northeast Fisheries Science Center Bottom Trawl Survey Data (OBIS-USA) | 48 | 1948 | 2008 | 36.62513 | -72.636 | 1023 | 439452 | NMFS. “Northeast Fisheries Science Center Bottom Trawl Survey Data (OBIS-USA).” NOAA’s National Marine Fisheries Service (NMFS) Northeast Fisheries Science Center. Woods Hole, Massachusetts, USA. 2005;Available at: http://www.iobis.org/mapper/?dataset=1435:accessed 2013. |
| 214 | Terrestrial | Temperate | Terrestrial plants | Long-term growth mortality and regeneration of trees in permanent vegetation plots in the Pacific Northwest 1910 to present | 88 | 1910 | 2010 | 45.34296 | -122.799 | 39 | 37350 | Harmon MF, J. “Long-term growth, mortality and regeneration of trees in permanent vegetation plots in the Pacific Northwest, 1910 to present.” Long-Term Ecological Research. Forest Science Data Bank, Corvallis. 2012;Available at: http://andrewsforest.oregonstate.edu/data/abstract.cfm?dbcode=TV010:accessed 2012. |
| 215 | Terrestrial | Temperate/Tropical | Birds | Hawk Migration Association of North America (HMANA) | 57 | 1952 | 2008 | 38.40865 | -99.5157 | 39 | 991769 | HMANA. "Hawk Migration Association of North America (HMANA)".Available at: http://www.hmana.org/:accessed 2012. |
| 217 | Terrestrial | Temperate | Birds | Landbird Monitoring Program (UMT-LBMP) | 14 | 1992 | 2006 | 46.82889 | -109.982 | 268 | 336516 | USFS. “Landbird Monitoring Program (UMT-LBMP).” US Forest Service.Available at: http://www.avianknowledge.net/:accessed 2012. |
| 219 | Terrestrial | Temperate | Amphibians | Marsh Monitoring Program - Amphibian Surveys | 17 | 1995 | 2011 | 44.90047 | -84.7768 | 14 | 41914 | NatureCounts. Bird Studies Canada "Marsh Monitoring Program." NatureCounts, a node of the Avian Knowledge Network. 2012;Available at: http://www.birdscanada.org/birdmon/:accessed 2012. |
| 220 | Terrestrial | Temperate | Birds | Marsh Monitoring Program - Bird Surveys | 17 | 1995 | 2011 | 46.88548 | -80.0332 | 243 | 144779 | NatureCounts. Bird Studies Canada "Marsh Monitoring Program." NatureCounts, a node of the Avian Knowledge Network. 2012;Available at: http://www.birdscanada.org/birdmon/:accessed 2012. |
| 221 | Terrestrial | Temperate | Terrestrial plants | Vegetation Plots of the Bonanza Creek LTER Control Plots Species Count (1975 - 2004) | 26 | 1975 | 2008 | 64.84423 | -148.052 | 52 | 1157 | Viereck LA, Van Cleve K, Chapin FS, Ruess RW, Hollingsworth TN. Vegetation Plots of the Bonanza Creek LTER Control Plots: Species Count (1975 - 2004). Environmental Data Initiative. 2005;Available at: http://dx.doi.org/10.6073/pasta/8dd0e1ac48e2f82b51adabfbd3c62ae2:accessed 2012. |
| 225 | Terrestrial | Temperate | Birds | Point count bird censusing long-term monitoring of bird distrubution and diversity in central Arizona-Phoenix period 2000 to 2011 | 12 | 2000 | 2011 | 33.43 | -111.93 | 278 | 48841 | Shochat E, Katti M, Warren P. “Point count bird censusing: long-term monitoring of bird distribution and diversity in central Arizona-Phoenix: period 2000 to 2011”. Central Arizona-Phoenix Long-Term Ecological Research. Global Institute for Sustainability, Arizona State University. 2004;Available at: https://caplter.asu.edu/data/data-catalog/?id=46:accessed 2012. |
| 229 | Freshwater | Temperate | Fish | Upper Little Tennessee River Biomonitoring Program Database - LTWA Biomonitoring Database | 26 | 1988 | 2013 | 35.13816 | -83.3855 | 69 | 11184 | McLarney WO, Meador J, Chamblee J. “Upper Little Tennessee River Biomonitoring Program Database.” Coweeta Long Term Ecological Research Program. 2010;Available at: https://coweeta.uga.edu/dbpublic/dataset_details.asp?accession=4045:accessed 2012. |
| 232 | Marine | Polar/Temperate | Fish | Pelagic Fish Observations 1968-1999 | 25 | 1968 | 1999 | -56.8147 | 93.88436 | 185 | 6446 | Williams D. “Pelagic Fish Observations 1968-1999.” Australian Antarctic Data Centre.Available at: http://www.gbif.org/dataset/85b0a82a-f762-11e1-a439-00145eb45e9a:accessed 2012. |
| 236 | Freshwater | Temperate | Fish | Fish population on selected watersheds at Konza Prairie - CFP012 - Konza fish population | 12 | 1995 | 2006 | 39.0931 | -96.5586 | 19 | 1169 | Gido KB. “Fish population on selected watersheds at Konza Prairie - CFP01.” Konza Prairie LTER Program.Available at: http://www.konza.ksu.edu/KNZ/pages/data/Knzdsdetail.aspx?datasetCode=CFP01:accessed 2012. |
| 240 | Terrestrial | Temperate | Terrestrial plants | Pinon-Juniper (Core Site) Quadrat Data for the Net Primary Production Study at the Sevilleta National Wildlife Refuge New Mexico (2003-present ) | 13 | 2003 | 2015 | 34.35 | -106.88 | 167 | 15561 | Muldavin E. "Pinon-Juniper (Core Site) Quadrat Data for the Net Primary Production Study at the Sevilleta National Wildlife Refuge, New Mexico (2003-Present)." Sevilleta Long Term Ecological Research Program.;Available at: http://sev.lternet.edu/node/1718:accessed 2013. |
| 243 | Terrestrial | Temperate | Terrestrial plants | Long-term N-fertilized vegetation plots on Hog Island Virginia Coastal Barrier Islands 1992 to 2014 | 22 | 1992 | 2014 | 37.44663 | -75.6675 | 51 | 8508 | Day F. “Long-term N-fertilized vegetation plots on Hog Island, Virginia Coastal Barrier Islands, 1992-2014.” Virginia Coast Reserve Long-Term Ecological Research Project. 2010;Available at: http://www.vcrlter.virginia.edu/cgi-bin/showDataset.cgi?docid=knb-lter-vcr.106:accessed 2013.  Day FP, Conn C, Crawford E, Stevenson M. Long-term effects of nitrogen fertilization on plant community structure on a coastal barrier island dune chronosequence. Journal of Coastal Research. 2004:722-30. |
| 244 | Marine | Temperate | Birds | British Columbia Coastal Waterbirds Survey | 14 | 1999 | 2012 | 50.55543 | -126.284 | 243 | 219126 | NatureCounts. Bird Studies Canada "BC Coastal Waterbird Survey (2004)." NatureCounts, a node of the Avian Knowledge Network. 2012;Available at: http://www.birdscanada.org/birdmon/:accessed 2012. |
| 246 | Marine | Temperate | Fish | Long-term monitoring dataset of fish assemblages impinged at nuclear power plants in northern Taiwan | 15 | 2000 | 2014 | 25.24431 | 121.6243 | 335 | 3722 | Chen H, Liao Y-C, Chen C-Y, Tsai J-I, Chen L-S, Shao K-T. Long-term monitoring dataset of fish assemblages impinged at nuclear power plants in northern Taiwan. Scientific data. 2015;2:150071. |
| 249 | Terrestrial | Temperate | Terrestrial invertebrates | Resource specialists lead local insect community turnover associated with temperature - analysis of an 18-year full-seasonal record of moths and beetles | 24 | 1992 | 2015 | 55.70251 | 12.55896 | 1427 | 31787 | Thomsen PF, Jørgensen PS, Bruun HH, Pedersen J, Riis-Nielsen T, Jonko K, *et al*. Resource specialists lead local insect community turnover associated with temperature – analysis of an 18-year full-seasonal record of moths and beetles. Journal of Animal Ecology. 2016;85(1):251-61. |
| 252 | Marine | Temperate | Fish | MARMAP Blackfish Trap Survey 1990-2009 | 13 | 1977 | 1989 | 32.45388 | -78.9682 | 48 | 4692 | Reichert M. “MARMAP Blackfish Trap Survey 1990-2009”. SCDNR/NOAA MARMAP Program. SCDNR MARMAP Aggregate Data Surveys. The Marine Resources Monitoring. Assessment. and Prediction (MARMAP) Program. Marine Resources Research Institute. South Carolina Department of Natural Resources USA. 2010;Available at: http://www.usgs.gov/obis-usa/:accessed 2013. |
| 253 | Freshwater | Temperate | Freshwater invertebrates | North Temperate Lakes LTER Zooplankton - Trout Lake Area 1982 - current | 29 | 1986 | 2014 | 46.02139 | -89.6529 | 133 | 30750 | LTER N. “North Temperate Lakes LTER: Zooplankton - Trout Lake Area 1982 - current.” NorthTemperate Lakes Long Term Ecological Research Program, Center for Limnology, University of Wisconsin-Madison.Available at: https://lter.limnology.wisc.edu/dataset/north-temperate-lakes-lter-zooplankton-trout-lake-area-1982-current:accessed 2013. |
| 256 | Marine | Temperate | Fish | ICES Beam Trawl Survey for commercial fish species. ICES Database of trawl surveys (DATRAS) - The Netherlands | 24 | 1987 | 2010 | 56.34138 | 2.672616 | 120 | 37250 | DATRAS. "Fish trawl survey: ICES Beam Trawl Survey for commercial fish species. ICES Database of trawl surveys (DATRAS)." The International Council for the Exploration of the Sea, Copenhagen. 2010;Available at: http://www.emodnet-biology.eu/data-catalog?%3Fmodule=dataset&dasid=2761:accessed 2013. |
| 271 | Marine | Temperate | Fish | Santa Barbara Coastal LTER | 15 | 2000 | 2014 | 34.30565 | -119.875 | 62 | 6287 | Reed DC. “SBC LTER: Reef: Kelp forest community dynamics: Abundance and size of giant kelp (Macrocystis pyrifera), ongoing since 2000”. Santa Barbara Coastal LTER. 2014a;Available at: http://sbc.lternet.edu/cgi-bin/showDataset.cgi?docid=knb-lter-sbc.18 doi:10.6073/pasta/d90872297e30026b263a119d4f5bca9f:accessed 2016. |
| 272 | Marine | Temperate | Marine invertebrates | Santa Barbara Coastal LTER | 15 | 2000 | 2014 | 34.30554 | -119.876 | 36 | 5363 | Reed DC. “SBC LTER: Reef: Kelp forest community dynamics: Fish abundance”. Santa Barbara Coastal LTER. 2014b;Available at: http://sbc.lternet.edu/cgi-bin/showDataset.cgi?docid=knb-lter-sbc.17 doi:10.6073/pasta/e37ed29111b2fddffc08355252b8b8c7:accessed 2016. |
| 273 | Marine | Temperate | Marine invertebrates | Santa Barbara Coastal LTER | 15 | 2000 | 2014 | 34.30554 | -119.876 | 27 | 15498 | Reed DC. “SBC LTER: Reef: Kelp forest community dynamics: Invertebrate and algal density”. Santa Barbara Coastal LTER. . 2014c;Available at: http://sbc.lternet.edu/cgi-bin/showDataset.cgi?docid=knb-lter-sbc.19 doi:10.6073/pasta/cd4cf864efecd69891dfe1d73b9ac9c3:accessed 2016. |
| 288 | Marine | Temperate | Fish | DFO Maritimes Research Vessel Trawl Surveys Fish Observations (OBIS Canada) | 34 | 1970 | 2006 | 43.97739 | -63.682 | 195 | 19074 | Clark D, Branton B. DFO Maritimes Research Vessel Trawl Surveys, OBIS Canada Digital Collections. Bedford Institute of Oceanography, Dartmouth, Nova Scotia, Canada, OBIS Canada. 2007. |
| 297 | Marine | Tropical | Marine invertebrates | MCR LTERCoral Reef Long-term Population and Community Dynamics Other Benthic Invertebrates. ongoing since 2005 | 11 | 2005 | 2015 | -17.5246 | -149.837 | 13 | 2734 | Carpenter R. “MCR LTER: Coral Reef: Long-term Population and Community Dynamics: Other Benthic Invertebrates, ongoing since 2005”. Moorea Coral Reef LTER, knb-lter-mcr.7.28. 2015;Available at: doi:10.6073/pasta/8e7b3a0c7a8bf315739921861cc79d10:accessed 2016. |
| 300 | Terrestrial | Temperate | Terrestrial invertebrates | Insect Populations via Sticky Traps at KBS-LTER (Kellogg Biological Station. MI) | 25 | 1989 | 2013 | 42.40885 | -85.3832 | 21 | 47798 | Landis D, Gage S. Insect Populations via Sticky Traps at KBS-LTER. 2014;Available at: http://lter.kbs.msu.edu/datatables/67:accessed 2016. |
| 301 | Terrestrial | Temperate | Terrestrial invertebrates | Konza LTER grasshopper monitoring. Konza Prairie LTER. KS | 25 | 1982 | 2013 | 39.106 | -96.611 | 51 | 10470 | Joern A. CGR02 Sweep Sampling of Grasshoppers on Konza Prairie LTER watersheds (1982-present). Environmental Data Initiative. 2016;Available at: http://dx.doi.org/10.6073/pasta/7060b2c244229a37e3bfc8c18f14ad02:accessed 2016.  Jonas JL, Joern A. Grasshopper (Orthoptera: Acrididae) communities respond to fire, bison grazing and weather in North American tallgrass prairie: a long-term study. Oecologia. 2007;153(3):699-711. |
| 308 | Terrestrial | Temperate | Mammals | Powdermill Nature Reserve monitored small mammal populations from 1979-1999. | 21 | 1979 | 1999 | 40.17074 | -79.2602 | 14 | 35398 | Merritt J. Long Term Mammal Data from Powdermill Biological Station 1979-1999. Environmental Data Initiative. 1999;Available at: http://dx.doi.org/10.6073/pasta/83c888854e239a79597999895bb61cfe:accessed 2016. |
| 309 | Terrestrial | Temperate | Terrestrial invertebrates | Monitoring the Abundance of Butterflies 1976-1985 | 10 | 1976 | 1985 | 53.13218 | -2.17768 | 44 | 5694 | Pollard E, Hall ML, Bibby TJ. Monitoring the Abundance of Butterflies 1976-1985. Research & survey in nature conservation. 1986;Available at: http://jncc.defra.gov.uk/page-2614:accessed 2016. |
| 311 | Terrestrial | Temperate | Mammals | Seasonal summary of numbers of small mammals on 14 LTER traplines in prairie habitats at Konza Prairie | 33 | 1981 | 2013 | 39.08333 | -96.5833 | 15 | 2458 | Kaufman DW. Seasonal summary of numbers of small mammals on 14 LTER traplines in prairie habitats at Konza Prairie. Konza Prairie Long-Term Ecological Research. .Available at: http://lter.konza.ksu.edu/content/csm01-seasonal-summary-numbers-small-mammals-14-lter-traplines-prairie-habitats-konza:accessed 2016. |
| 313 | Terrestrial | Temperate | Terrestrial invertebrates | Successional Dynamics on a Resampled Chronosequence Core Old Field Grasshopper Sampling | 18 | 1989 | 2006 | 45.4 | -93.2 | 61 | 7958 | Knops J, Tilman D. Successional Dynamics on a Resampled Chronosequence - Experiment 014. Cedar Creek Ecosystem Science Reserve. .Available at http://www.cedarcreek.umn.edu/research/data/dataset?ghe014:accessed 2016. |
| 316 | Terrestrial | Temperate | Reptiles | Lizard pitfall trap data (LTER-II LTER-III) | 18 | 1989 | 2006 | 32.62 | -106.74 | 21 | 2650 | Lightfoot D. “Lizard pitfall trap data (LTER-II, LTER-III)”. Jornada Basin LTER. 2013;Available at: http://jornada.nmsu.edu/lter/dataset/49821/view:accessed 2016. |
| 318 | Terrestrial | Temperate | All | Karoo National Park Census Data | 13 | 1994 | 2009 | -32.2333 | 22.28333 | 25 | 229 | SANParks. "Karoo National Park Census Data. 1994 - 2009". 2011;Available at: http://datadryad.org/handle/10255/dryad.13079?show=full:accessed 2016. |
| 319 | Terrestrial | Temperate | All | Effects of rangeland management on community dynamics of herpetofauna to the tallgrass prairie | 14 | 1989 | 2003 | 37.25 | -96.7167 | 35 | 232 | Wilgers DJ, Horne EA, Sandercock BK, Volkmann AW. Effects of rangeland management on community dynamics of the herpetofauna of the tallgrass prairie. Herpetologica. 2006;62(4):378-88. |
| 321 | Terrestrial | Temperate | Mammals | Small Mammal Exclosure Study. Jornada LTER. SMES rodent trapping data | 13 | 1995 | 2007 | 32.55034 | -106.812 | 19 | 12787 | Lightfoot D, Schooley RL. “SMES rodent trapping data, Small Mammal Exclosure Study”. Jornada LTER.Available at: http://jornada.nmsu.edu/sites/jornada.nmsu.edu/files/data_files/JornadaStudy_086_smes_rodent_trapping_data_0.csv:accessed 2016. |
| 327 | Terrestrial | Temperate | Mammals | Fray Jorge Small Mammals 1989-2005 | 17 | 1989 | 2005 | -30.6 | -71.7 | 12 | 256469 | Kelt D, Meserve P, Gutiérrez J, Milstead WB, Previtali M. Long‐term monitoring of mammals in the face of biotic and abiotic influences at a semiarid site in north‐central Chile. Ecology. 2013;94(4):977-. |
| 328 | Freshwater | Temperate | Amphibians | The Rainbow Bay Long-term Study | 30 | 1979 | 2008 | 32.26 | -81.63 | 10 | 301 | Scott D, Metts B, Lance S. “The Rainbow Bay Long-term Study”.Available at: http://srelherp.uga.edu/projects/rbay.htm:accessed 2016. |
| 332 | Freshwater | Temperate | Fish | Stream Fish Assemblage stability in a southern Appalachian stream (Coweeta Hydro Lab 1984 - 1995) | 12 | 1984 | 1995 | 35.0589 | -83.4319 | 14 | 590 | Grossman GD. “Stream fish assemblage stability in a southern Appalachian stream at the Coweeta Hydrologic Laboratory from 1984 to 1995”. Coweeta Long Term Ecological Research Program. 2007;Available at: http://dx.doi.org/10.6073/pasta/f0baf5f59c89f670e04f537f5cc05290:accessed 2016. |
| 336 | Terrestrial | Temperate | Terrestrial plants | Long term monitoring and experimental manipulation of a Chihuahuan Desert ecosystem near Portal Arizona | 14 | 1989 | 2002 | 31.93889 | -109.08 | 100 | 35978 | Ernest S, Valone TJ, Brown JH. Long‐term monitoring and experimental manipulation of a Chihuahuan Desert ecosystem near Portal, Arizona, USA. Ecology. 2009;90(6):1708-. |
| 339 | Terrestrial | Temperate | Birds | Species trends turnover and composition of a woodland bird community in southern Sweden during a period of 57 years. | 57 | 1953 | 2009 | 55.71667 | 13.33333 | 39 | 1210 | Svensson S, Thorner A, Nyholm N. Species trends, turnover and composition of a woodland bird community in southern Sweden during a period of fifty-seven years. Ornis Svecica. 2010;20(1):31-44. |
| 340 | Terrestrial | Temperate | Terrestrial plants | Small Mammal Exclosure Study (SMES) Vegetation Data from the Chihuahuan Desert | 15 | 1995 | 2009 | 34.296 | -106.927 | 93 | 1608 | Lightfoot D. “Small Mammal Exclosure Study (SMES) Vegetation Data from the Chihuahuan Desert Grassland and Shrubland at the Sevilleta National Wildlife Refuge, New Mexico (2006-2009)”. Long Term Ecological Research Network. 2011;Available at: http://dx.doi.org/10.6073/pasta/d80d5e2196cd11ef79df23ebe5a77c19:accessed 2016. |
| 348 | Terrestrial | Temperate/Tropical | Mammals | Bats (Mammalia Chiroptera) in restinga in the municipality of Jaguaruna south of Santa Catarina Brazil. | 10 | 2006 | 2016 | -28.6089 | -48.9813 | 13 | 177 | Carvalho F, Zocche JJ, Mendonça RÁ. Morcegos (Mammalia, Chiroptera) em restinga no município de Jaguaruna, sul de Santa Catarina, Brasil. Biotemas. 2009;22(3):193-201. |
| 356 | Terrestrial | Tropical | Terrestrial plants | Long-term stem inventory data from tropical rain forest plots in Australia | 34 | 1971 | 2013 | -17.0381 | 145.5612 | 478 | 14938 | Bradford MG, Murphy HT, Ford AJ, Hogan DL, Metcalfe DJ. Long‐term stem inventory data from tropical rain forest plots in Australia. Ecology. 2014;95(8):2362-. |
| 357 | Terrestrial | Temperate | Mammals | Small Mammal Trapping Webs on the Central Plains Experimental Range | 13 | 1994 | 2006 | 40.82889 | -104.758 | 10 | 1104 | Stapp P. SGS-LTER Long-Term Monitoring Project: Small Mammals on Trapping Webs on the Central Plains Experimental Range, Nunn, Colorado, USA 1994 -2006, ARS Study Number 118. Environmental Data Initiative. 2013;Available at: http://dx.doi.org/10.6073/pasta/2e311b4e40fea38e573890f473807ba9:accessed 2017. |
| 359 | Marine | Temperate | Fish | SBC LTER Reef Kelp Forest Community Dynamics Fish abundance | 13 | 2000 | 2012 | 34.30891 | -119.874 | 61 | 3285 | Reed DC. “SBC LTER: Reef: Kelp Forest Community Dynamics: Fish abundance”. Santa Barbara Coastal LTER. 2014;Available at: doi:10.6073/pasta/e37ed29111b2fddffc08355252b8b8c7:accessed 2016. |
| 361 | Terrestrial | Temperate | Birds | A long-term bird population study in an Appalachian spruce forest | 22 | 1962 | 1983 | 38.61 | -79.8347 | 20 | 213 | Hall GA. A long-term bird population study in an Appalachian spruce forest. The Wilson Bulletin. 1984:228-40. |
| 366 | Terrestrial | Temperate | Mammals | Small Mammal Exclosure Study (SMES) | 25 | 1989 | 2013 | 34.35 | -106.88 | 24 | 3389 | Lightfoot D. “Small Mammal Exclosure Study (SMES)”. Sevilleta Long Term Ecological Research Program.Available at: http://sev.lternet.edu/content/small-mammal-exclosure-study-smes-0:accessed 2016. |
| 374 | Marine | Temperate | Birds | Monitoring site 1000 Shorebird Survey | 11 | 2004 | 2014 | 35.96125 | 136.0461 | 70 | 38674 | Monitoring Site 1000 Project, Biodiversity Center, Japan MoEo. “Monitoring site 1000 Shorebird Survey” 2013;(ShorebirdsDatapackage2012.zip, downloaded from http://www.biodic.go.jp/moni1000/ findings/data/index.html):Accessed 2016. |
| 375 | Terrestrial | Temperate | Terrestrial invertebrates | Surface of the earth wandering beetles survey data | 11 | 2004 | 2014 | 36.24489 | 136.9066 | 424 | 19937 | Monitoring Site 1000 Project, Biodiversity Center, Japan MoEo. “Monitoring site 1000 Forest and grassland research - Surface wandering beetles survey data”. 2014;(GBDataPackage2014ver1.zip, downloaded from http://www.biodic.go.jp/moni1000/findings/data/index.html):Accessed 2016. |
| 378 | Marine | Temperate | Marine invertebrates | Calafuria Mid-shore Intertidal Dataset (1991-2014) | 16 | 1991 | 2006 | 43.46937 | 10.33596 | 42 | 1239 | Benedetti-Cecchi L. “Calafuria Mid-shore Intertidal Dataset (1991-2014)”. Department of Biology, University of Pisa.Accessed 2016. |
| 379 | Marine | Temperate | Marine invertebrates | Calafuria Low-shore Intertidal Dataset (1991-2014) | 22 | 1991 | 2014 | 43.46937 | 10.33596 | 61 | 1153 | Benedetti-Cecchi L. “Calafuria Mid-shore Intertidal Dataset (1991-2014)”. Department of Biology, University of Pisa.Accessed 2016. |
| 380 | Terrestrial | Temperate | Terrestrial invertebrates | Monitoring butterfly numbers | 10 | 1978 | 1987 | 50.8463 | 0.0552 | 23 | 217 | Pollard E. Monitoring butterfly numbers. In: F. B.Goldsmith (ed). Monitoring for Conservation and Ecology Chapman and Hall. 1991.  NERC. “The Global Population Dynamics Database Version 2”. Centre for Population Biology, Imperial College. 2010;Available at: http://www.sw.ic.ac.uk/cpb/cpb/gpdd.html:accessed 2016. |
| 382 | Terrestrial | Temperate | Mammals | Small Mammals and Vegetation Changes After Fire in a Mixed Conifer-Hardwood Forest | 13 | 1955 | 1967 | 47.83333 | -91.8333 | 7 | 214 | NERC. “The Global Population Dynamics Database Version 2”. Centre for Population Biology, Imperial College. 2010;Available at: http://www.sw.ic.ac.uk/cpb/cpb/gpdd.html:accessed 2016.  Krefting LW, Ahlgren CE. Small Mammals and Vegetation Changes After Fire in a Mixed Conifer‐Hardwood Forest. Ecology. 1974;55(6):1391-8. |
| 419 | Marine | Temperate | Birds | Data collected aboard cruises off the coast of the Western Antarctic Penninsula | 19 | 1993 | 2011 | -64.77 | -64.05 | 54 | 3050 | Fraser W. “At-sea seabird censuses. Data on the species encountered (including marine mammals), their abundance, distribution and behavior. Data collected aboard cruises off the coast of the Western Antarctic Peninsula, 1993 - present”. Palmer Station Antarctica LTER. 2014;Available at: http://dx.doi.org/10.6073/pasta/e3871e749fa737dd94d5a269ac90e8ce:accessed 2016. |
| 420 | Terrestrial | Polar/Temperate | Birds | Species composition and population fluctuations of alpine bird communities during 38 years in the Scandinavian mountain range | 38 | 1964 | 2001 | 67.077 | 17.435 | 47 | 1010 | Svensson S. Species composition and population fluctuations of alpine bird communities during 38 years in the Scandinavian mountain range. Ornis Svecica. 2006;16(4):183-210. |
| 428 | Marine | Temperate | All | Long term monitoring of fish abundances from coastal SKagerrak | 97 | 1919 | 2015 | 58.95856 | 9.768152 | 59 | 101221 | Olsen EM, Carlson SM, Gjøsæter J, Stenseth NC. Nine decades of decreasing phenotypic variability in Atlantic cod. Ecology Letters. 2009;12(7):622-31.  Rogers LA, Stige LC, Olsen EM, Knutsen H, Chan K-S, Stenseth NC. Climate and population density drive changes in cod body size throughout a century on the Norwegian coast. Proceedings of the National Academy of Sciences. 2011;108(5):1961-6.  Stenseth NC, Bjørnstadf ON, Falck W, Fromentin JM, Gjøsieter J, Gray JS. Dynamics of coastal cod populations: intra- and intercohort density dependence and stochastic processes. Proceedings of the Royal Society of London Series B: Biological Sciences. 1999;266(1429):1645-54.  Barceló C, Ciannelli L, Olsen EM, Johannessen T, Knutsen H. Eight decades of sampling reveal a contemporary novel fish assemblage in coastal nursery habitats. Global Change Biology. 2016;22(3):1155-67. |
| 430 | Freshwater | Temperate | Fish | The New Zealand Freshwater Fish Database - Electric fishing - Backpack | 32 | 1985 | 2016 | -40.9428 | 172.4993 | 57 | 22054 | NIWA. “The New Zealand Freshwater Fish Database”.Available at: https://www.niwa.co.nz/our-services/online-services/freshwater-fish-database:accessed 2016. |
| 431 | Freshwater | Temperate | Fish | The New Zealand Freshwater Fish Database - Traps- Gee Minnow traps | 33 | 1984 | 2016 | -40.6336 | 172.4499 | 44 | 3804 | NIWA. “The New Zealand Freshwater Fish Database”.Available at: https://www.niwa.co.nz/our-services/online-services/freshwater-fish-database:accessed 2016. |
| 432 | Freshwater | Temperate | Fish | The New Zealand Freshwater Fish Database - Observation (Spotlighting visual) | 19 | 1998 | 2016 | -40.4926 | 172.7156 | 36 | 3520 | NIWA. “The New Zealand Freshwater Fish Database”.Available at: https://www.niwa.co.nz/our-services/online-services/freshwater-fish-database:accessed 2016. |
| 441 | Terrestrial | Temperate | Birds | Long-term dynamics of bird populations in birch forests of Ilmen Nature Reserve during the breeding period individuals / km2 | 13 | 1985 | 1997 | 54.50408 | 60.294 | 61 | 384 | Zakharov VD. Biodiversity of bird population of terrestrial habitats in Southern Ural. Miass: IGZ. Ural Branch of Russian Academy of Sciences. 1998:158 p. |
| 466 | Marine | Temperate | Fish | Trawl Survey Data from Rockall Scotland (1986 - 2008) | 23 | 1986 | 2008 | 56.99054 | -9.07255 | 94 | 27592 | Neat F, Campbell N. Demersal fish diversity of the isolated Rockall plateau compared with the adjacent west coast shelf of Scotland. Biological Journal of the Linnean Society. 2011;104(1):138-47. |
| 471 | Terrestrial | Temperate | Terrestrial plants | Prescribed Burn Effect on Chihuahuan Desert Grasses and Shrubs at the Sevilleta National Wildlife Refuge | 10 | 2004 | 2013 | 34.35 | -106.88 | 162 | 37979 | Muldavin E, Collins SL. Prescribed Burn Effect on Chihuahuan Desert Grasses and Shrubs at the Sevilleta National Wildlife Refuge, New Mexico: Species Composition Study 2004 to present. Sevilleta LTER. 2003;Available at: http://sev.lternet.edu/data/sev-166:accessed 2016. |
| 473 | Terrestrial | Temperate | Terrestrial plants | Fourteen years of mapped permanent quadrats in a northern mixed prairie | 14 | 1932 | 1945 | 46.31667 | -105.8 | 95 | 2350 | Anderson J, Vermeire L, Adler PB. Fourteen years of mapped, permanent quadrats in a northern mixed prairie, USA. Ecology. 2011;92(8):1703-. |
| 475 | Terrestrial | Temperate | Birds | Structure and dynamics of a passerine bird community in a spruce-dominated boreal forest | 12 | 1960 | 1972 | 63.41667 | 10.5 | 34 | 327 | Hogstad O, editor Structure and dynamics of a passerine bird community in a spruce-dominated boreal forest. A 12-year study. Annales Zoologici Fennici; 1993: JSTOR. |
| 477 | Marine | Temperate | Marine invertebrates | Epifaunal invertebrate survey from Goodwin Islands York River Estuary Chesapeake Bay | 15 | 1998 | 2012 | 37.222 | -76.392 | 41 | 5914 | Douglass JG, France KE, Richardson JP, Duffy JE. Seasonal and interannual change in a Chesapeake Bay eelgrass community: Insights into biotic and abiotic control of community structure. Limnology and Oceanography. 2010;55(4):1499-520.  Lefcheck JS. The use of functional traits to elucidate the causes and consequences of biological diversity. The College of William & Mary, PhD thesis. 2015;Available at: http://gradworks.umi.com/36/62/3662989.html:accessed 2016. |
| 478 | Freshwater | Temperate | Freshwater invertebrates | Long term study of the stream ecosystems in the Breitenbach | 37 | 1969 | 2005 | 50.66972 | 9.628889 | 90 | 1537 | Wagner R, Marxsen J, Zwick P, Cox EJ. Central European Stream Ecosystems: The Long Term Study of the Breitenbach: John Wiley & Sons; 2011 |
